# Acute Ethanol-Induced Changes in Edema and Metabolite Concentrations in Rat Brain

**DOI:** 10.1155/2014/351903

**Published:** 2014-03-25

**Authors:** Huimin Liu, Wenbin Zheng, Gen Yan, Baoguo Liu, Lingmei Kong, Yan Ding, Zhiwei Shen, Hui Tan, Guishan Zhang

**Affiliations:** ^1^Department of Radiology, The Second Affiliated Hospital of Shantou University Medical College, Shantou, Guangdong 515041, China; ^2^Department of Ultrasound, The Affiliated Yuebei People's Hospital of Shantou University Medical College, Shaoguan, Guangdong 512025, China; ^3^Department of Neurosurgery, The Affiliated Yuebei People's Hospital of Shantou University Medical College, Shaoguan, Guangdong 512025, China

## Abstract

The aim of this study is to describe the acute effects of EtOH on brain edema and cerebral metabolites, using diffusion weight imaging (DWI) and proton magnetic resonance spectroscopy (^1^H-MRS) at a 7.0T MR and to define changes in apparent diffusion coefficient (ADC) values and the concentration of metabolites in the rat brain after acute EtOH intoxication. ADC values in each ROI decreased significantly at 1 h and 3 h after ethanol administration. ADC values in frontal lobe were decreased significantly compared with other regions at 3 h. For EtOH/Cr+PCr and cerebral metabolites (Cho, Tau, and Glu) differing over time, no significant differences for Ins, NAA, and Cr were observed in frontal lobes. Regression analysis revealed a significant association between TS_EtOH/Cr+PCr _and TS_Cho_, TS_Tau_, TS_Glu_, and TS_ADC_. The changes of ADC values in different brain regions reflect the process of the cytotoxic edema in vivo. The characterization of frontal lobes metabolites changes and the correlations between TS_EtOH/Cr+PCr _and TS_Cho_, TS_Tau_, and TS_Glu_ provide a better understanding for the biological mechanisms in neurotoxic effects of EtOH on the brain. In addition, the correlations between TS_EtOH/Cr+PCr_ and TS_ADC_ will help us to understand development of the ethanol-induced brain cytotoxic edema.

## 1. Introduction

The consumption of ethanol (EtOH) and the subsequent production of its oxidative metabolites affect both developing and mature nervous systems, directly or indirectly, and have acute and chronic complications [1]. In particular, acute EtOH intake has pronounced effects on brain metabolism and the cell functional and morphologic changes. In vivo characterization of brain metabolites and the changes of the cell function can readily be performed by proton magnetic resonance spectroscopy (^1^H-MRS) and diffusion weight imaging (DWI). After EtOH administration, the cerebral metabolite levels of ethanol (EtOH), N-acetyl-aspartate (NAA), choline-containing compounds (Cho), glutamate (Glu), taurine (Tau), inositol (Ins), and creatine (Cr) and the classification of the brain edema can be reliably assessed, especially under the high-field MRI.

In previous studies, most magnetic resonance imaging (MRI) investigations mostly report chronic effects of EtOH on regional brain structure and brain metabolites [2–4]. However, there are few imaging data on acute EtOH effects on brain function and metabolites.

Regarding the effects of chronic EtOH intake on the brain structure, several magnetic resonance imaging (MRI) studies using conventional structural magnetic resonance imaging (MRI) and diffusion tensor imaging (DTI) report reduced volume of both gray matter and white matter in the cerebral cortex. In addition some studies have explored the most prominent changes in brain volume which have been reported for the frontal cortex and the cerebellum.

For chronic EtOH effects, most ^1^H-MRS studies report reduced levels of NAA and Cho compared with control subjects [4–7]. Most studies have revealed no effects of EtOH on cerebral Cr levels. In addition, findings of EtOH effects on cerebral Ins may be depressed [8]. In animal model, investigation of the chronic ethanol exposure showed no significant effects of EtOH oncerebral NAA and Cho [8].

Compared with NAA, Cr, and Cho, however, in vivo quantification of Glu, Tau is challenging at low-field strengths MR scanner. A number of comparison studies show that precision of metabolite quantification and detectability of weakly represented metabolites are substantially increased at 7T relative to low-field in vivo ^1^H-NMR spectroscopy [9].

The purpose of this study was to delineate the acute impact of EtOH on the brain using DWI and ^1^H-MRS at a 7.0T MR scanner. Emphasis was placed on the assessment of the changes of ADC values and the concentration of brain metabolites. A specific cascade of statistical analyses was applied for the ADC values and every metabolite concentration time series. In addition, We hypothesize that the change of the EtOH level in the frontal lobe detected by ^1^H-MRS was correlated with ADC values in early acute EtOH intoxication, which will be useful for better understanding of the biological mechanisms in neurotoxic effects of EtOH on the brain.

## 2. Materials and Methods

### 2.1. Animals

Sixty sexually mature (aged 8–10-week-old) male Sprague-Dawley rats (weight ranged from 200 to 250 g) were used for the experiments according to a protocol approved by the Animal Care Committee at Shantou University Medical Center. The rats were maintained under conditions of constant temperature, humidity, and 12-hour light/dark cycles. Rats were treated in compliance with NIH guidelines for the care and use of laboratory animals. After one week of stabilization on an ad lib diet, rats were randomly divided into DWI group and ^1^H-MRS group. Thirty rats in DWI group and ^1^H-MRS group were then randomly divided into five acute EtOH intoxication groups (1 h, 3 h, 6 h, 12 h, 24 h) and one control group. Rats in the acute EtOH intoxication groups were given ethanol (56% v/v) via an orogastric tube. As previously described [10], the total ethanol dosage for one acute EtOH intoxication rat was 15 mL/kg body wt. For the control group, animals were given 15 mL/kg of drinking water by gastric administration. Anesthesia was induced with 10% chloral hydrate for a total dose of 3 mL/kg by intraperitoneal injection before MRI examination.

### 2.2. MRI Acquisition

All MR experiments were performed on an Agilent 7.0 Tesla animal MR scanner (Agilent VnmrJ 3 Imaging, USA). Animals were positioned in a cradle and the head was placed directly on a 3 cm radiofrequency surface coil. A 3-plane localizer scan was acquired to ensure the animal brain was in the proper position. Conventional MRI, DWI, and ^1^H-MRS were separately performed in the DWI group and ^1^H-MRS group. The rats in the five subgroups (1 h, 3 h, 6 h, 12 h, and 24 h) of acute EtOH intoxication were separately imaged at 1 h, 3 h, 6 h, 12 h, and 24 h after EtOH administration.

Conventional images were obtained with T2(FSE 2500/48)-weighted spin echo sequences (both with a 192 × 192 matrix, 1 mm slice thickness, and 4 averages). Conventional images were assessed for the presence of normal anatomy and abnormal signal intensities separately and before diffusion analysis.

### 2.3. DWI Experiments and Data Processing

DWI was acquired using a fast spin echo multislice. Twenty slices of 1 mm thickness were obtained (repetition time 3500 ms, echo time 36 ms, field of view 3.6 cm,* b* values of 0 and 1000 s /mm^2^, and 8 averages) in axial directions. The total acquisition time was 24 min. DWI and ADC maps were separately calculated with diffusion weighting in three orthogonal directions, averaged over directions (mean ADC). DWI and ADC maps were visually assessed for abnormal signal intensity, consistent with restricted diffusion and therefore probable tissue edema. ADC values for region of interest (ROI) measurements were obtained independently by two observers. ROIs included frontal lobes, hippocampi, thalamus, and cerebellum. Measurements were made for both right and left sides of the brain.

### 2.4. ^1^H-MRS Regions of Interest and Data Processing

The chosen voxel of interest (VOI) was localized based on a series of SCOUT (TR 102 ms, TE 12 ms) coronal and sagittal images. A voxel (3 × 6 × 3 mm^3^) was located on the frontal lobe region using a stimulated echo acquisition mode (STEAM) sequence (TR/TE: 5000/2 ms). Magnetic field homogeneity was optimized by shimming on the water signal using the FASTMAP method. Water suppression was achieved with a preset pulse of the STEAM sequence.

Spectroscopic data were postprocessed by a Linear Combination of Model (LCModel) and a Java-based magnetic resonance user interface (jMRUI). The concentrations of NAA, Cho, Cr, Glu, Tau, and Ins were obtained using LCModel for automatic estimation of the metabolite concentrations. Then the concentrations of ethanol (EtOH) were expressed as EtOH/Cr+PCr using jMRUI.

### 2.5. Statistical Analysis

Analyses were undertaken using SPSS 13.0. Statistical data postprocessing encompassed the following subsequent analysis steps. (1) For each brain region, ADC value changes over time were tested for significance, using repeated measurements analyses of variance (rmANOVA). The method of Bonferroni was used to do pairwise comparisons of the repeatedly measured ADC values in different measurement time of each brain region. With multivariate ANOVA, ADC values in different region of each measurement time could be compared pairwise. (2) For each metabolite, concentration changes over time were tested for significance, using repeated measurements analyses of variance (rmANOVA). Metabolites concentration and ADC values in frontal lobe significantly changing after EtOH intake were supplied to a linear regression analysis to estimate associations between time series metabolite concentration (TSmet), TS_ADC_ and TS_EtOH/Cr+PCr_. *P* values < 0.05 were considered significant.

## 3. Results

### 3.1. Diffusion-Weighted Imaging

**Figure 1:**
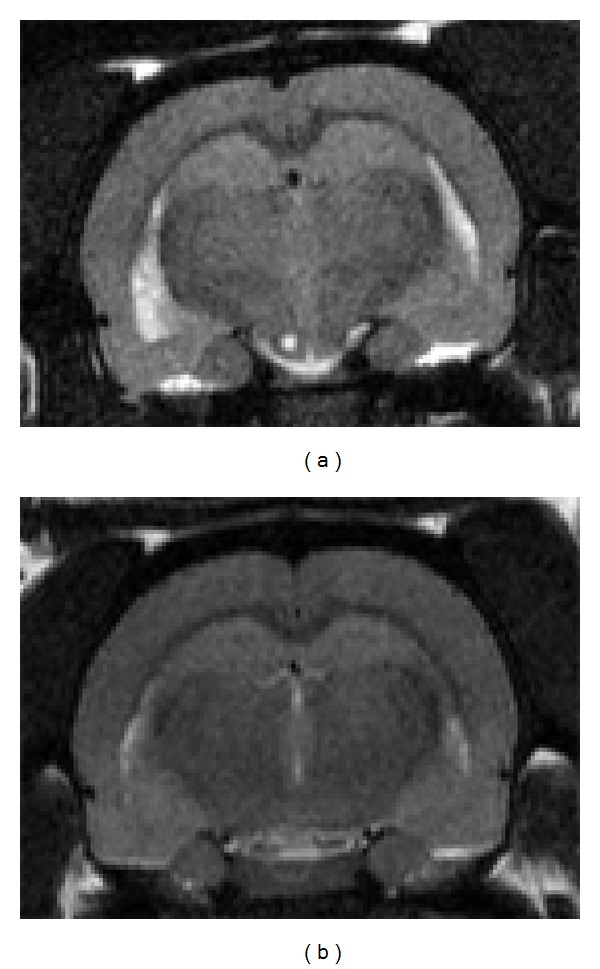
T2WI of the rat hippocampi ((a): control group; (b): 3 h).

**Figure 2:**
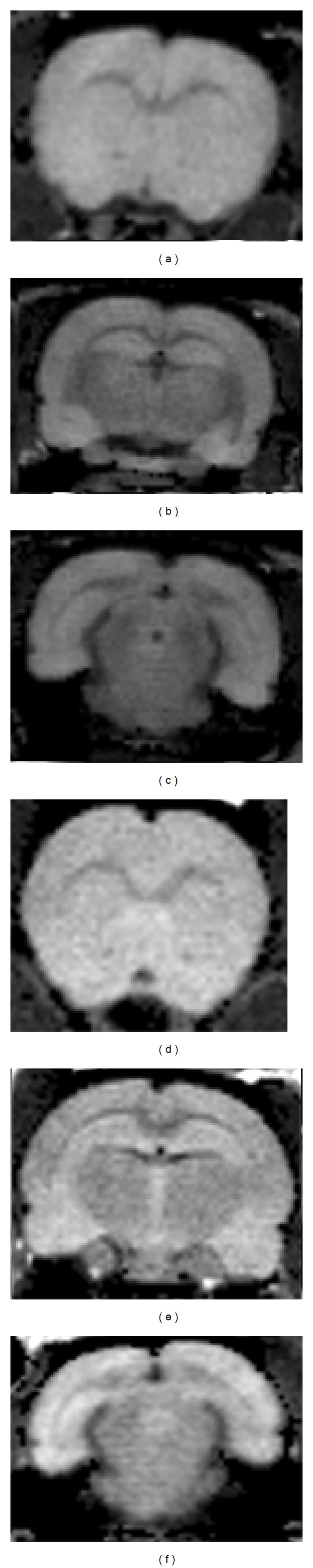
DWI maps of control and 3 h after EtOH exposure. There is hyperintensity on the DWI images in 3 h. (2(a)–2(c): control group; 3(a)–3(c): 3 h after EtOH exposure).

**Figure 3:**
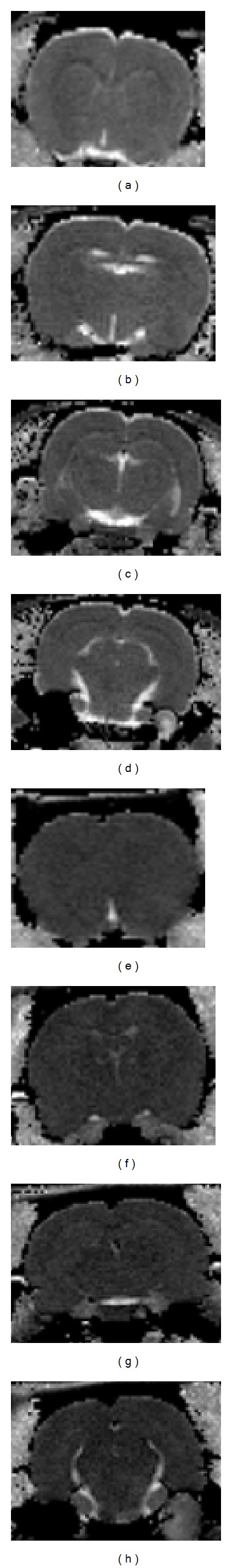
ADC maps of control and 3 h after EtOH exposure. There is hypointensity on the ADC images in 3 h. (3(a)–3(d): control group; 3(e)–3(f): 3 h after EtOH exposure).

**Figure 4:**
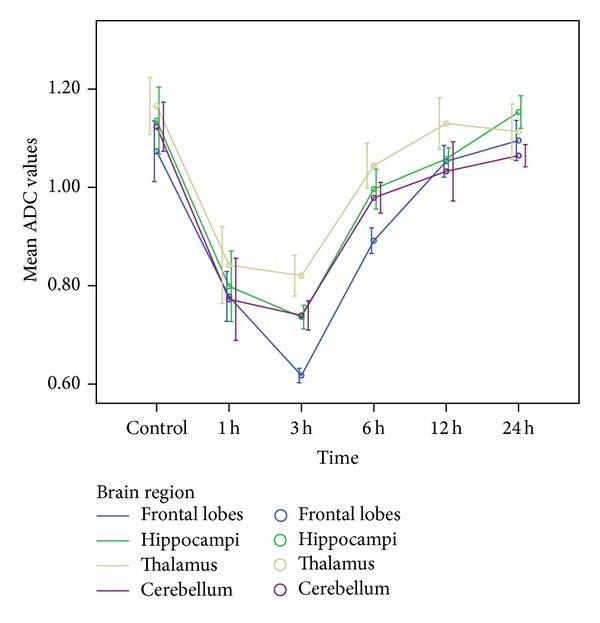
Changes of ADC values at different time points in four rat brain regions.

No signal intensity abnormalities were detected on the T2WI (Figure 1) of all rats. DWI and apparent diffusion coefficient (ADC) maps were calculated. DWI maps showed hyperintensity in the acute intoxication (Figure 2), and ADC images identified lower signal intensity than the control group (Figure 3). ADC value changes over time were significant in four brain regions (rmANOVA, *P* < 0.01). ADC values for the four brain regions were all significantly different at 1 h and 3 h after EtOH intake compared with the control group. The ADC values for the four brain regions are shown in Table 1. Compared with the control group, ADC values decreased beginning at 1 h, reached the minimum value at 3 h, and then increased gradually afterwards (Figure 4). ADC values in the frontal lobe were lower than other regions at 3 h (*P* < 0.05). In addition, there was a significant reduction compared with thalamus.

### 3.2. ^1^H-MRS

**Figure 5:**
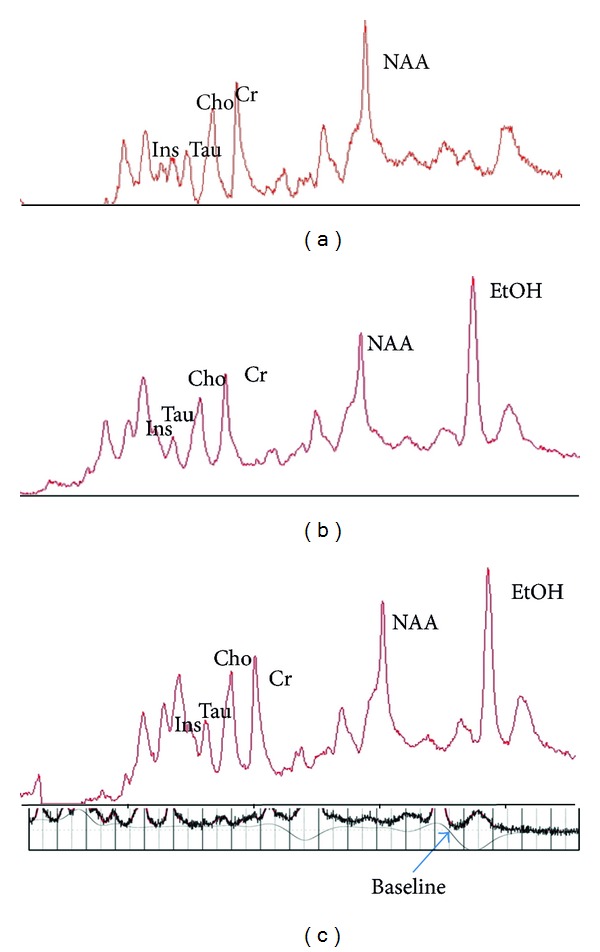
^1^H-magnetic resonance 7T spectroscopic spectra acquired from prefrontal lobes in the control (5a) and at 1 h (5b), 3 h (5c) after EtOH exposure.

**Figure 6:**
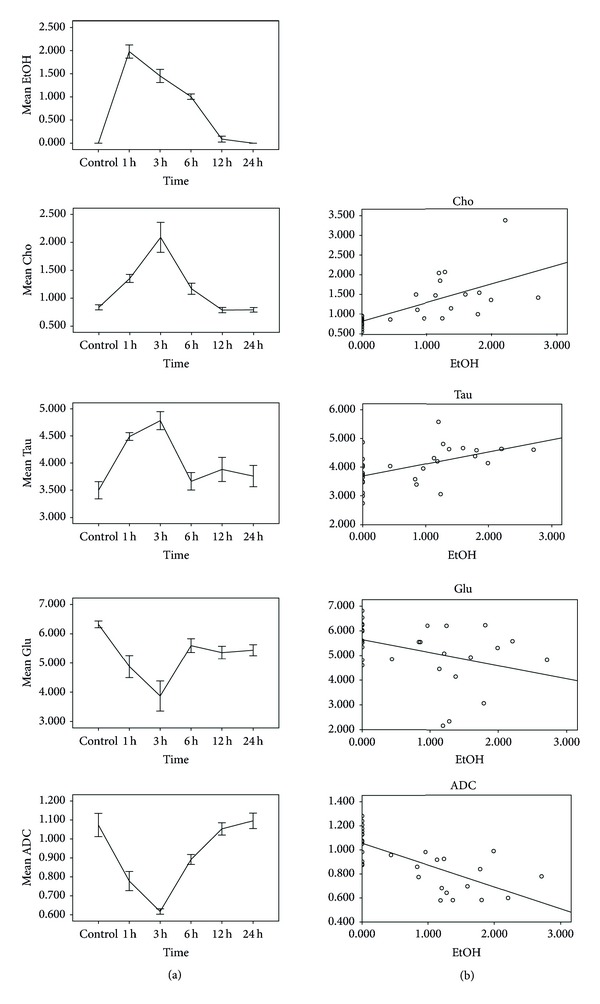
EtOH/tCr+PCr, cerebral metabolites, and ADC value. Left column: upon EtOH exposure, frontal lobe Cho, Tau, Glu levels, and ADC value differed over time; s.d. depicted as error bars. Right column: regression analysis revealed a significant association between TS_EtOH/Cr+PCr_ and TS_Cho_, TS_Tau_ (positive) and between TS_EtOH/Cr+PCr_ and TS_Glu_, TS_ADC_ (negative).

Figure 5 shows ^1^H-magnetic resonance spectroscopic spectra acquired from frontal lobes in control and 1 h, 3 h groups after EtOH exposure. At 1.18 and 3.67 ppm, the main resonance of the EtOH was clearly detectable after EtOH exposure. The measured data of the EtOH concentrations was expressed EtOH/Cr+PCr. EtOH/Cr+PCr changes over time were significant (rmANOVA; *P* < 0.01) (Table 2). EtOH/Cr+PCr increased in the frontal lobe, reaching the peak at 1 h and decreasing gradually due to clearance at 12 h (Figure 6).

Upon EtOH application, ADC values changed over time in frontal lobe (rmANOVA, *P* < 0.01). Regression analysis showed a negative correlation between TS_ADC_ and TS_EtOH/Cr+PCr_ (*P* < 0.01).

After EtOH exposure, Cho, Tau, and Glu concentrations of the frontal lobe changed over time (rmANOVA, *P* < 0.05) (Table 2). Regression analysis showed a positive correlation between TS_Cho_ and TS_EtOH/Cr+PCr_ (*P* < 0.01). TS_Tau_ was positively correlated with TS_EtOH/Cr+PCr_ (*P* < 0.01). For Glu, regression analysis showed a negative correlation between TS_Glu_ and TS_EtOH/Cr+PCr_ (*P* < 0.05) (Table 2) (Figure 6).

For Ins, NAA, Cr+PCr, and rmANOVA revealed no change over time after EtOH exposure (Table 2).

## 4. Discussion

Diffusion imaging is both a quantitative and sensitive method that provides a direct view of molecular displacement in tissues. The changes of ADC value reflect the extracellular space and the intracellular volumes. Generally diffusion of molecules within the intracellular volume is less limited than diffusion of molecules in the intercellular space. Many investigators believe that restricted water mobility is seen in lesions with cytotoxic edema, whereas increased water mobility is seen in lesions with vasogenic edema. Cell swelling accompany with decreases in the ADC of various molecules due to diffusion is hindered by the size of the extracellular clefts, the presence of membrane [11]. The Na^+^/K^+^-ATPase requires 0.5–4 h to reach a minimum [10]. We show that during this time, the major extent of severe brain cytotoxic edema occurs. ADC values in the frontal lobe, hippocampi, thalamus, and cerebellum decrease by 1 h and gradually reach a minimum value at 3 hour after EtOH ingestion. ADC maps show hypointensity diffusely scattered throughout the brain, reflecting a decrease in the molecular motion of water, consistent with accumulation of water within the intracellular space. This suggests development of cytotoxic edema within 3 h after EtOH consumption. On the contrary, after 3 h postacute EtOH intoxication, increases in ADC maybe suggest development of vasogenic edema. The changes of ADC value in our study correspond to actual pathophysiological mechanisms of cytotoxic brain edema after acute exposed to EtOH [10], which contradicted to the early study [12]. There are several possible explanations for the contradictory findings. First, ethanol doses used for inducing intoxication in rats in Rooney et al. study were much lower than that in our study. Second, they evaluated the effects of acute ethanol administration using the T_1_ relaxation times, to our knowledge, the changes of ADC value are more sensitive to demonstrate water molecular displacement in tissues. In addition, the T_1_ measurement in the Rooney et al. [12] report located in white matter.

Different cognitive-behavioral abilities are differentially sensitive to ethanol [13]. Frontal lobes play a major role in cognitive function, such as attention, working memory, creative and critical thinking, planning, decision making, inhibitory control, and emotional regulation [14]. Frontal lobe pathology in EtOH intoxication has been well documented and studied at the neurophysiological, morphological, and neuropsychological levels [15]. In our study, ADC values in the frontal lobe are lower than other regions at 3 h. This frontal lobe reduction is particularly significant compared with the thalamus and other regions and reflects a greater vulnerability of frontal lobes to the effects of acute EtOH consumption.

Various studies have explored the kinetics of EtOH metabolism and the effects of EtOH on cerebral metabolism [10, 16, 17]. Previous studies showed that about 40–80 min is the time when peak EtOH concentrations are reached in the brain after administration [17]. At 80–140 min after intake, ethanol concentrations show a slight decrease, almost reaching a steady state, indicating ethanol diffuses into the brain parenchyma to reach a new equilibrium [18]. The concentration of EtOH in frontal lobe detected in our ^1^H-MRS study shows a similar pattern. After 3 h postacute EtOH consumption, ethanol concentrations display a drop due to clearance, followed by a combination of absorption, metabolism, and elimination. We also find that EtOH levels significantly affects choline, taurine, and glutamate concentrations in the frontal lobes, and EtOH/tCr correlates well with these metabolite levels.

Phosphocholine, glycerophosphocholine, and free choline are grouped as Cho, a substrate for the synthesis of cell membranes and neurotransmitters [19] and highly concentrated in glial cells [20]. Some studies have shown that Cho is increased during acute consumption of EtOH, demonstrating the adaptation of brain phospholipid membrane to the ethanol [21]. Our results reveal increased Cho in the frontal lobes, suggesting that increased choline levels may reflect increased turnover of phosphatidylcholine and other phospholipids as an adaptive mechanism of the brain.

Taurine is an aminosulfonic acid that plays protective roles against neurochemical impairment induced by ethanol. Several reports demonstrate that Tau plays pivotal roles in central nervous system homeostasis, acting on regulation of osmotic pressure, antioxidation, neuromodulatory processes, and inhibitory neurotransmission. Our results show that Tau levels are increased at 1 h. Increased Tau levels have been reported in the frontal cortex, nucleus accumbens under acute and chronic ethanol administration in both rats and mice [22, 23]. Results from previous studies and our own study suggest that the Tau ratio levels may be an important indicator in EtOH intoxication.

Glu is the major excitatory neurotransmitter. Microdialysis studies report that levels of glutamate decrease with higher doses of EtOH, suggesting that EtOH may suppress glutamatergic transmission [24–26]. Prior investigations suggest that the reduction of Glu neurotransmission in the brain may be involved in cognitive deficits associated with high intoxicating doses of EtOH. In our study, the effect of high EtOH doses on the levels of glutamate is in agreement with previous in vitro studies that have shown a reduction in glutamatergic transmission at higher intoxicating concentrations [24].

Findings of EtOH effects on cerebral NAA, Ins levels are highly ambiguous, ranging from reduction to elevation [4, 7, 27–29]. Cr is generally considered to be a stable metabolite; many studies apply Cr as reference for relative metabolite quantification. For this study, we found NAA, Ins, and Cr of no significant change before versus after EtOH ingestion.

In conclusion, our results suggest that the increase in mean diffusivity found in different brain regions reflect the process of cytotoxic edema in vivo. The characterization of frontal lobe metabolites after acute EtOH intoxication can readily be performed by proton magnetic resonance spectroscopy (1H-MRS). The correlation of TS_EtOH/Cr+PCr_ and TS_ADC_ values, TS_Cho_, TS_Tau_ and TS_Glu_ suggest EtOH can induce and affect development of cell swelling by EtOH influx and mediating osmolyte channels and metabolite changes. Thus DWI and ^1^H-MRS experiments focusing on neuroimaging might offer a broad understanding for the neurotoxic effects of EtOH, especially in the cytotoxic edema.

A few potential limitations of the current study should be examined. First, this work mainly focuses on the frontal lobe, which seems to be more vulnerable to the effects of acute alcohol consumption as revealed by DTI. Other regions should be investigated. Second, our experimental designs could not use the ^1^H-MRS and DTI sequence in the same rat group because the scan period was too long to be unaffected by the clearance of the ethanol concentrations in each scan time point. Future studies exploring the relationship between structure and metabolite levels in a longitudinal design will be valuable.

## Figures and Tables

**Table 1:** ADC values for all groups in four brain regions (*x* ± *s*).

ADC value (×10^−3^)	Frontal lobe	Hippocampus	Thalamus	Cerebellum
control	1.073 ± 0.185	1.135 ± 0.205	1.166 ± 0.175	1.123 ± 0.151
1 h	0.778 ± 0.153*	0.799 ± 0.217*	0.842 ± 0.236*	0.772 ± 0.252*
3 h	0.701 ± 0.075**	0.736 ± 0.073**	0.820 ± 0.124**	0.740 ± 0.089**
6 h	0.892 ± 0.079	0.997 ± 0.122	1.044 ± 0.138	0.979 ± 0.094
12 h	1.053 ± 0.098	1.058 ± 0.068	1.130 ± 0.159	1.033 ± 0.182
24 h	1.095 ± 0.123	1.153 ± 0.100	1.113 ± 0.171	1.065 ± 0.068

*P < 0.05 was considered to indicate a statistically significant difference.

**P < 0.01 was considered to indicate an obviously statistically significant difference.

**Table 2:** Repeated measurements ANOVA of frontal lobe metabolite concentrations and ADC values.

	rmANOVA	Regression analysis			
	*P*	*P*	*β*/*R*	*F*	*R* ^2^
EtOH/Cr + PCr	0.000	NA	NA	NA	NA
Cho	0.000	0.000	0.669	21.834	0.447
Tau	0.005	0.003	0.539	11.031	0.290
Glu	0.018	0.042	−0.381	4.574	0.145
Ins	0.065 (NS)	NA	NA	NA	NA
Cr + PCr	0.165 (NS)	NA	NA	NA	NA
NAA	0.081 (NS)	NA	NA	NA	NA
ADC_FC_	0.000	0.000	−0.716	29.383	0.512

NA: not applied; NS: not significant; rmANOVA: repeated measurements analyses of variance.
